# P-1409. Molecular Detection of Tubercular Lymphadenitis: Real World Evidence for a Novel PCR-Based Assay

**DOI:** 10.1093/ofid/ofaf695.1596

**Published:** 2026-01-11

**Authors:** Nihar Ranjan Nayak, Sandeep Rao Kordcal, Pankaj Jorwal, Prayas Sethi, Animesh Ray, Manish Soneja, Naveet Wig

**Affiliations:** All India Institute of Medical Sciences, New Delhi, New Delhi, Delhi, India; All India Institute of Medical Sciences, New Delhi, Delhi, India; All India Institute of Medical Sciences, New Delhi, New Delhi, Delhi, India; All India Institute of Medical Sciences, New Delhi, New Delhi, Delhi, India; All India Institute of Medical Sciences, New Delhi, New Delhi, Delhi, India; All India Institute of Medical Sciences, New Delhi, Delhi, India; All India Institute of Medical Sciences, New Delhi, New Delhi, Delhi, India

## Abstract

**Background:**

Tubercular lymphadenitis (LNTB) is the most common form of extrapulmonary tuberculosis (EPTB). GeneXpert Ultra, a cartridge-based nucleic acid amplification test, offers advancements over its predecessor, GeneXpert, including a larger reaction chamber and two additional multi-copy amplification target genes, promising to improve diagnostic accuracy. We conducted a two-year prospective observational study to evaluate the diagnostic accuracy of GeneXpert Ultra in 55 LNTB patients.

**Table 1.**
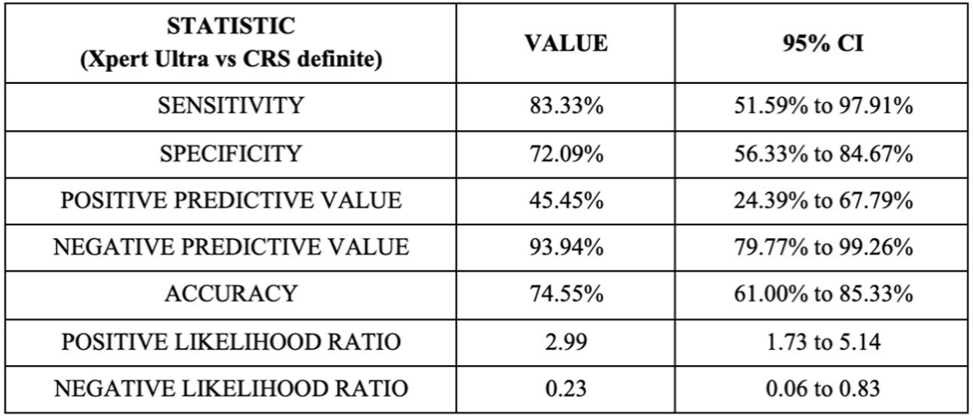
Diagnostic performance of LN GeneXpert Ultra against CRS : definite LNTB

**Table 2.**
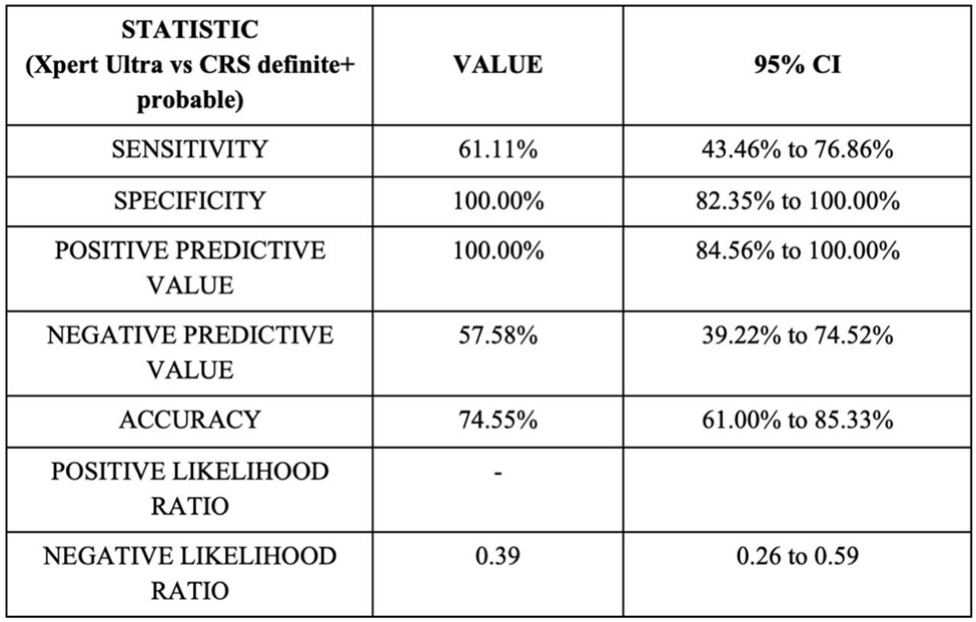
Diagnostic performance of LN GeneXpert Ultra against CRS: probable+definite LNTB

**Methods:**

Participants underwent lymph node biopsy and samples obtained were subjected to Mycobacteria Growth Indicator Tube (MGIT) culture, Ziehl-Neelsen staining, and cytology/histopathology. Diagnostic accuracy of GeneXpert Ultra was assessed against a composite reference standard (CRS) incorporating clinical, histopathological, and microbiological findings. Sensitivity, specificity, positive predictive value (PPV), and negative predictive value (NPV) of Ultra were calculated against both CRS and MGIT culture

**Results:**

Fifty-five participants were included (mean age 33.1 years; 55% female). Ultra was positive in 22 (40%) cases. Against MGIT culture, Ultra showed a sensitivity of 100%, specificity of 64.7%, PPV of 18.2%, and NPV of 100%. However, MGIT culture positivity was low (7.2%), consistent with prior studies reporting low culture yields in lymph node tuberculosis due to paucibacillary samples. Therefore, diagnostic performance was primarily assessed against CRS. Ultra showed a sensitivity of 61.1% (95% CI 43.5–76.9) and specificity of 100% (95% CI 82.3–100), against CRS (confirmed + probable LNTB), with a PPV of 100% and an NPV of 57.6%.When restricted to CRS-confirmed LNTB cases alone, Ultra demonstrated a sensitivity of 83.3% (95% CI 51.6–97.9) and specificity of 72.1% (95% CI 56.3–84.7)

**Conclusion:**

GeneXpert MTB/RIF Ultra demonstrated high specificity and moderate overall sensitivity for diagnosing lymph node tuberculosis, with improved sensitivity among microbiologically confirmed cases. Despite low MGIT culture positivity, Ultra outperformed culture in case detection and represents a valuable diagnostic tool in paucibacillary tubercular lymphadenitis when interpreted alongside clinical and histopathological findings.

**Disclosures:**

All Authors: No reported disclosures

